# Chiropractic Care in Children: A Review of Evidence and Safety

**DOI:** 10.1177/00099228241305202

**Published:** 2024-12-22

**Authors:** Sanghamitra M. Misra, Omar Jaber, Caroline Long

**Affiliations:** 1 Department of Pediatrics, Baylor College of Medicine, Houston, TX, USA; 2 Texas Children’s Hospital, Houston, TX, USA; 3 Children’s National Pediatricians, Children’s National Hospital, Washington, DC, USA; 4 School of Medicine & Health Sciences, The George Washington University, Washington, DC, USA; 5 West University Wellness, Bellaire, TX, USA

**Keywords:** chiropractic care, integrative medicine, integrative pediatrics, spinal manipulative therapy

## Abstract

Complementary therapies are used to treat many pediatric symptoms and health conditions, and chiropractic care is one of the most commonly used forms of complementary therapies by children and adolescents. Research studies have investigated the evidence behind and safety of chiropractic care in pediatrics with various musculoskeletal and non-musculoskeletal conditions. There are limited data with a range of findings and often no definite conclusion. Despite the paucity of evidence of benefits of chiropractic care in children, the considerations around safety, and the differing opinions regarding pediatric chiropractic practice inside and outside the field, many pediatric patients visit chiropractors, and chiropractors often care for pediatric patients. Pediatric health care providers should discuss the use of all complementary therapies with patients, so guidance can be optimal with a focus on promoting health and safety.

## Background

Complementary therapies are used to treat many childhood symptoms and health conditions.^1^ Chiropractic care including spinal manipulative therapy (SMT) is a very commonly used pediatric complementary therapy.^2,3^ According to a 2012 US national study, prevalence of chiropractic use in children was 3.5%, with use higher among adolescents, Midwest US residents, families with income ≥$100 000, and those who visited other complementary and alternative medicine (CAM) practitioners.^4^ The 2017 update to the same US national study showed that the prevalence was essentially unchanged with 3.4% (2 million children) utilizing chiropractic care, adolescents were more likely to have seen a chiropractor (5.1%) than younger children ages 4 to 11 years (2.1%), and children with mental health issues were using chiropractic care (5.3%).^5,6^ In 1 outpatient general pediatrics clinic, 19% of children visited a chiropractor.^7^ Among children with inborn errors of metabolism, 1 study showed that 41% used chiropractic care.^8^ Although it may seem intuitive that patients visit chiropractors for musculoskeletal problems, studies have investigated and reported benefits in children with non-musculoskeletal conditions such as upper respiratory and ear infections, autism, infantile colic, and attention deficit/hyperactivity disorder (ADHD). Children represent 8% to 15% of all chiropractic visits, and the top 5 reasons for use of chiropractic care include wellness care, ear-nose-throat issues, digestive issues, musculoskeletal problems, ADHD, and headaches.^9,10^ Children with neurological conditions utilize complementary therapies more often than children without neurological diseases, and chiropractic care is the most commonly used complementary therapy in this population.^11-13^

## General Evidence

Although chiropractic care has been minimally researched in pediatrics, there have been multiple published systematic reviews concluding that there is limited quality and low supporting evidence of the effectiveness of chiropractic care in children and adolescents. One of those examined spinal manipulative therapy for headaches and/or mechanical spinal pain (4 studies included in review) and 1 examined chiropractic manipulation (57 studies included in review).^1,14^ Multiple literature reviews have concluded that chiropractic care and SMT for non-musculoskeletal disorders could not be proven or disproven for pediatric patients.^15,16^ A different systematic review found no evidence of an effect of chiropractic treatment for primary or secondary prevention of non-musculoskeletal diseases and warned chiropractors to not misrepresent chiropractic care in areas where there is no scientific evidence for its use.^17^ When chiropractors consider a treatment that has no evidence for effectiveness, they should inform the patients and their parents, but this is not routinely done.^18^ A 2019 review of 50 pediatric manual therapy studies including chiropractic concluded some effectiveness for lower back pain, nursemaid’s elbow, and breastfeeding, but no improvement for torticollis.^19^ A systematic review of 26 SMT studies in children and adolescents showed very little evidence of effectiveness and data collection focused on parent and patient perceptions.^20^ In a 2012 review article including studies on asthma, autism, breastfeeding, colic, ear infections, enuresis, and jet lag, studies that used specific outcome measures commonly relevant to families concluded higher effectiveness, and effectiveness data for children with asthma were the strongest.^21^ Most studies of chiropractic care have significant limitations, and most reasons for chiropractic treatment in children have not yet been proven to be effective or ineffective.^22^

### Asthma

Chiropractors theorize that due to the mechanics of thoracic cage restriction, SMT improves asthma symptoms by decreasing or completely eliminating thoracic cage restriction, and this effect can improve patients’ perception of symptoms.^23,24^ However, a review of 8 studies concluded that although children and adults with asthma may feel benefit from chiropractic care, we do not have enough scientific data proving its effectiveness, so chiropractic care should never be used in place of standard medical treatments.^25^

### Attention Deficit/Hyperactivity Disorder

It has been theorized that chiropractic adjustments may improve ADHD symptoms by enhancing prefrontal cortex function. Although SMT may be feasible for these children, there is little evidence of efficacy supporting the use of chiropractic care for children and adolescents with ADHD. In 1 randomized controlled study, there was no difference in chiropractic adjustment vs sham chiropractic in children.^26,27^

### Autistic Spectrum Disorders

Some chiropractors theorize that exchange of sensory information between neurons is disturbed in autism, and chiropractic care can improve that communication.^28^ A 2011 systematic literature review concluded that there was insufficient research available to determine the effectiveness of chiropractic care for treatment of autism in children.^29^

### Breastfeeding

Chiropractic care aims to help infants with breastfeeding difficulties by correcting cervico-cranio-mandibular dysfunction, but there is inconclusive evidence for the use of SMT or craniosacral therapy for breastfeeding.^19,30^

### Colic

In a review of 6 infantile colic studies, 5 suggested beneficial effects with statistical significance for parent report of fewer hours of crying daily, but 1 study concluded that SMT provided no benefit. There were limitations in most of the colic studies such as potential bias since parents assessing the crying were not blinded to the intervention. If analyzing only studies with low risk of bias, improvement in crying was not statistically significant.^31^ In a subsequent 2021 single-blind randomized controlled trial from Denmark including 185 infants, duration of crying was 1.5 hours in the treatment group and 1 hour in the control group, but when adjusting for all cofounders, the difference was not statistically significant.^32^ At this time, there is insufficient safety data on SMT for infantile colic.^31^

### Headache and Back Pain

A review of 166 pediatric articles on SMT for back pain and headaches concluded low levels of evidence for chiropractic manipulation. A 2021 study of almost 200 children found that SMT resulted in less frequent headaches and perceived global effects, but it did not lower headache severity.^33^ A study of 51 adolescent and young adult patients demonstrated that a course of chiropractic care including SMT is a reasonable pain management option.^34^

### Otitis Media

Chiropractors hypothesize that since abnormalities in the cervical spine may result in Eustachian tube dysfunction and subsequent disruption of lymphatic drainage, so chiropractors may use lymph drainage techniques to relieve pressure.^35^ Children suffering from otitis media (OM) may benefit from SMT, but a 2012 review of 49 studies concluded that there is not enough evidence to recommend chiropractic care for OM.^36^

### Respiratory Illness

In a 2013 review article, 6 of 8 studies showed some benefit from manual therapies in chronic respiratory illnesses such as asthma and cystic fibrosis.^37^

### Immunizations

Some chiropractors may oppose mandatory immunizations because their education is based on Palmer’s theory, which rejects the germ theory of disease in favor of a theory of spinal subluxation as the cause of disease.^38^ The International Chiropractic Association states that families should have the choice whether or not to immunize their children.^39^

## Safety

Many pediatricians are concerned about the safety of chiropractic care in their patients. There are little data on adverse events from chiropractic care, but serious adverse events are rare.^40,41^ Unlike the high-velocity, low-amplitude thrust manipulations used with adults, most techniques used in pediatric patients are gentle, low-force, and appear to be safe.^42^ According to a 2015 review study of 31 articles, serious adverse events in infants and children receiving chiropractic care are rare, and no deaths had been reported.^43^ However, significant adverse events have been documented with chiropractic care in pediatric patients such as subarachnoid hemorrhage, recurrent stroke, paraplegia, severe headache, and midback soreness as well as delays of diagnosis and inappropriate use of chiropractic care for severe illness.^44-46^

## Ethics

The ethics of using chiropractic care in children is complex, particularly due to the lack of robust scientific evidence regarding its safety and efficacy. Ethical considerations must prioritize every child’s well-being, ensuring that care is based on the best available evidence. However, since there is very limited funding for chiropractic care research, robust studies are rarely performed. Informed consent is crucial. Parents need clear communication of potential risks, benefits, and alternatives. Using unproven treatments in children may risk harm, whether physical or by diverting resources away from more effective interventions. In addition, the fact that some chiropractors hold anti-vaccine views adds another ethical layer, as it can undermine public health efforts and influence parents away from proven, life-saving interventions. However, respecting parents’ autonomy in making health care decisions for their children is important, provided that they are fully informed of the evidence and possible outcomes. Pediatric health care professionals must weigh all these factors carefully as they guide their patients and families.

## Conclusion

Despite the paucity of evidence of benefit, the considerations around safety, and the differing opinions regarding the appropriateness of pediatric chiropractic practice inside and outside the field, patients see chiropractors, and chiropractors provide care to children. Pediatricians must discuss the use of chiropractic care and all complementary therapies with patients, so guidance and treatment of every pediatric patient can be safe and optimal.

## Author Contributions

**SMM:** Drafted, critically reviewed, and revised the manuscript; approved the final manuscript as submitted and agree to be accountable for all aspects of the work.

**OJ:** Drafted, critically reviewed, and revised the manuscript; approved the final manuscript as submitted and agree to be accountable for all aspects of the work.

**CL:** Drafted, critically reviewed, and revised the manuscript; approved the final manuscript as submitted and agree to be accountable for all aspects of the work.
